# Synthesis and Characterization of Nanodiamond Reinforced Chitosan for Bone Tissue Engineering

**DOI:** 10.3390/jfb7030027

**Published:** 2016-09-15

**Authors:** Yu Sun, Qiaoqin Yang, Haidong Wang

**Affiliations:** Department of Mechanical Engineering, University of Saskatchewan, Saskatoon, SK S7N 5A9, Canada; yus441@mail.usask.ca (Y.S.); wanghaidong.wang@usask.ca (H.W.)

**Keywords:** nanodiamond, chitosan, scaffold, nanoindentation, DSC, MTT

## Abstract

Multifunctional tissue scaffold material nanodiamond (ND)/chitosan (CS) composites with different diamond concentrations from 1 wt % to 5 wt % were synthesized through a solution casting method. The microstructure and mechanical properties of the composites were characterized using scanning electron microscopy (SEM), X-ray diffraction (XRD), Fourier transform infrared spectroscopy (FTIR), and nanoindentation. Compared with pristine CS, the addition of ND resulted in a significant improvement of mechanical properties, including a 239%, 276%, 321%, 333%, and 343% increase in Young’s modulus and a 68%, 96%, 114%, 118%, and 127% increase in hardness when the ND amount was 1 wt %, 2 wt %, 3 wt %, 4 wt %, and 5 wt %, respectively. The strong interaction between ND surface groups and the chitosan matrix plays an important role in improving mechanical properties.

## 1. Introduction

Tissue engineering has shown great potential in treatment of injured tissues including bone tissues. Statistically, bone injuries occur at an annual rate of 2.4 per 100 population [1]. In the United States, there were over 2 million bone injuries in 2005, costing $17 billion. By 2025, annual bone injuries and costs are projected to increase by 50% and $25 billion, respectively [2]. The major therapy used for clinical treatments of bone repair and regeneration, to date, is conventional bone grafting. Annually in the United States, over half a million patients receive bone defect repairs [3]. However, problems still persist due to disease transmission and limited supply. Engineered bone tissue, as an alternative approach to treating bone injuries, has shown great potential for bone injury treatment by creating an artificial structure to repair and regenerate damaged and diseased bone tissues [4]. Nevertheless, its clinical application is limited by several challenges, one of which is designing a biodegradable and biocompatible scaffold with sufficient mechanical strength that is comparable with natural bone [5].

Chitosan (CS) is a natural polysaccharide, obtained from the deacetylation of chitin which is the primary structural polymer of the exoskeleton of crustaceans, cuticles of insects, and cell wall of fungi [6,7]. The CS market is expected to be 4.22 billion in 2020 [8]. As more innovative applications have been approved in recent years by the FDA for commercializing patents, CS will be more extensively used, especially in biomedical areas. In acidic environments, CS can be dissolved completely in acid solution which makes it feasible to be produced in the shapes of any required structure: microspheres, membranes, sponges, fibers, and 3D porous, etc. which are suitable for scaffold constructions [9]. From the viewpoint of biological properties, cationic nature of CS allows electrostatic interactions with anionic glycosaminoglycans (GAGs) and proteoglycans. When protonated CS carries a high value of positive charge, it interacts with the negative surface of living tissues such as proteins and nucleic acids to obtain electrical neutrality [10]. As a promising scaffold material for bone tissue engineering, CS has demonstrated essential features including biodegradability and biocompatibility [11,12,13]. Previous research indicated CS could be a potent wound-healing accelerator [9], cytokines producer [14], and can inhibit infection [15]. Abundance of free amino groups also allows various chemical modifications for specific purposes. Nevertheless, the poor strength of pure CS does not match the physical demands of the healthy surrounding bone and may not provide a stable structure for cell growth as bone scaffolds. Ideally, the scaffold should be provided with enough mechanical strength for surgical handling during implantation and support the load that the bone would sustain until the natural bone can regenerate at the site of surgery. The mechanical properties of bone vary widely with density. Cancellous bone, as an example, has a 50–500 MPa modulus which can serve as a design goal for bone regeneration.

It is noted that even though ceramics, synthetic and natural materials have been well developed and modified for bone tissue engineering, it is still difficult for a single material to meet all requirements in terms of mechanical, biological and functional properties. This necessitates considerable research in developing composite scaffolds comprising a number of phases as novel materials for bone tissue engineering to address the issue mentioned above. Composite materials are aimed at imitating the composite of natural bone and obtain a combined effect to eliminate the disadvantages of individual material, of which polymer based nanocomposite materials are very promising. To date, much effort has been made in developing and designing such composites with fillers in nanometer scale. Of the recent promising hybrid materials, the nanocomposite system with nanoscale ceramic fillers and polymer matrix has shown a great improvement in performance because the nanosized inorganic component is likely to be more bioactive than a micro-sized one. Fillers in nanoscale, when interacted with polymer matrix, result in a larger surface area. When the nanofillers interact with the polymer matrix, a new interphase forms. If this interphase is strong, the load and heat will be transferred from the polymer matrix to the nanofillers. Smaller-sized fillers will result in a larger specific surface area, which means that the contact area between polymer matrix and fillers is larger. The interphase plays a crucial role in determining the properties of the composites. Appropriate selection of matrix and fillers along with certain treatment can significantly increase the quality of the interphase and thus lead to a great improvement in mechanical and thermal properties. Besides, due to high surface area to volume ratio, highly porous scaffolds with exceptional mechanical properties can be fabricated with a wide variety of topographical features that encourage cell adhesion and proliferation. Nano fillers are incorporated into polymer matrices at rates from 1% to 10% (in mass) [16]. A perfect candidate filler in polymer matrix for biomedical applications must have numerous properties: high strength and stiffness, resistance to corrosion in natural hazardous environment, nontoxicity, biocompatibility and low costs.

NDs, with the outstanding properties of super hardness, high Young’s modulus, superior thermal conductivity and biocompatibility, are considered as a very promising material for various applications in the fields of chemistry, optical spectroscopy, physical and mechanical engineering, biological and biomedical engineering [17]. NDs produced by detonation are composed of nano-scale particles at a diameter of around 5 nm. An ND particle consists of an inert diamond core terminated with rich surface functional groups. The chemical reactivity on the surface allows a variety of wet and gas chemistry techniques to be employed to tailor the properties of NDs for specific purposes, such as biomolecules attachment for drug delivery [18], microelectromechanic devices [19] and cardiovascular devices [20]. The primary core of detonation ND is sp^3^ hybridized carbon. By sharing carbon-based composition with many biological components, ND is stable and presents a biocompatible interface with no generation of reactive oxygen species (ROS) including oxygen ions and peroxides which are chemically reactive molecules containing oxygen [21]. It has also been claimed that ND can be internalized at a variety of concentrations by individual cells and expelled through the body’s circulatory system [22]. Due to these features, ND has been studied for biomedical applications as a composite additive, a drug delivery carrier, a multiple radical donor, implants and coating materials [23]. Studies have been done to investigate various kinds of ND/polymer (PVA, PC, PMMA, epoxy, and PLA) nanocomposites. A significant improvement in the tensile modulus and fracture energy has been achieved by the addition of a low percentage of ND [24,25,26,27,28,29]. However, No ND/CS composites have been reported up to now. In the present work, we choose ND as a filler for CS matrix because of its non-cytotoxicity and modification potential compared with most other filler materials. To fully benefit from the uniqueness of ND, functionalization is the key to utilizing ND for certain applications. Both wet and gas chemical treatment can be applied to modify its surface [30].

This research aimed to synthesize a functional composite material with desirable properties including biocompatibility, biodegradability and enhanced mechanical strength for bone scaffold by introducing nanodiamond (ND) fillers to the chitosan (CS) matrix, and an innovative approach to fabricating ND/CS composites, which uses nanodiamonds as reinforcement filler to improve the mechanical properties of chitosan, is reported. Furthermore, the interaction between CS and ND and the structural, mechanical properties of the composites were investigated. The results have demonstrated that the composites are very promising for use as bone scaffolds to improve mechanical properties. As we described in the previous paragraphs, the biggest challenge to synthesizing composites with improved mechanical properties is to disperse nanodiamonds into the CS matrix. We adopted an appropriate functionalization approach combined with sonication to address the challenge and the composites synthesized demonstrated significantly improved mechanical properties. 

## 2. Materials and Methods

### 2.1. Materials

CS powder (β-1,4-linked N-acetyl-D-glucosamine (GlcNAc) with degree of deacetylation (DD) of approximately 80% were purchased from Sigma-Aldrich Canada and used as received. The viscosity is less than 200 mPa.s and contains 1% in acetic acid (20 °C). Explosion synthesized ND powders were purchased from Nanostructured and Amorphous Materials, Inc. with an average particle size of 5 nm and purity over 95%.

Dulbecco’s modified Eagle Medium (DMEM) and MTT (3-4,5-dimethylthiazol-2yl{-2,5-diphenyl-2Htetrazolium bromide) were purchased from Sigma–Aldrich (St. Louise, MO, USA). Foetal bovine serum (FBS) was purchased from Invitrogen Co, Carlsbad, CA, USA. NaOH (sodium hydroxide) and other reagents used were all of analytical grade.

Phosphate buffered saline (PBS) was prepared by dissolving pre-determined amount of reagent-grade NaCl (90 mg/mL), NaH_2_PO_4_·H_2_O (0.31725 mg/mL) and Na_2_HPO_4_·7H_2_O (2.064 mg/mL) (Sigma-Aldrich, St. Louis, MO, USA) in DIW. To be specific, 1.269 g NaH_2_PO_4_·H_2_O, 8.256 g Na_2_HPO_4_·7H_2_O and 360 g NaCl were weighed with an analytical balance, respectively, and dissolved in 4 L deionized water.

### 2.2. ND Functionalization

ND has a large specific surface area and rich functional groups. In particular, the high density of carboxylic and hydroxyl groups on the surface of ND facilitates interaction with the CS matrix. Considering the major interaction between the carboxylic groups on the ND surface and CS polymer chains [31,32], the as-received ND was treated by concentrated acid to increase the concentration of the carboxylic group, as well as to remove the surface impurities introduced from the manufacturing process such as oxides and carbides, including those of iron, chromium, silicon, calcium, copper, potassium, titanium, and sulfur, in addition to carbon soot [33], which may impact the properties of CS polymers. Detailed steps are described in the following paragraph. Such functionalized NDs were used as precursors for further characterization and experiments.

Five grams of as-received ND powder was treated with 150 mL of concentrated acid solution of HNO_3_ (70%) and H_2_SO_4_ (98%) at a volume ratio of 1:3 [34], and sonicated (2510 BRANSON Sonicator) for 1 h at room temperature for an adequate dispersion. Subsequently, the solution was heated at 60 °C for 12 h to remove impurities and oxidize surface groups. After the acid treatment, functionalized ND particles were applied to a centrifugation (Allegra X-22R Centrifuge, BECKMAN COULTER) of 3000 r/min for 15 min and rinsed with deionized water three times. The cycles were repeated until the supernatant became pH neutral. The resultant product was dried in a freeze dryer overnight for 8 h to yield purified carboxyl functionalized ND powders.

### 2.3. Preparation of CS/ND Composites

**Figure 1:**
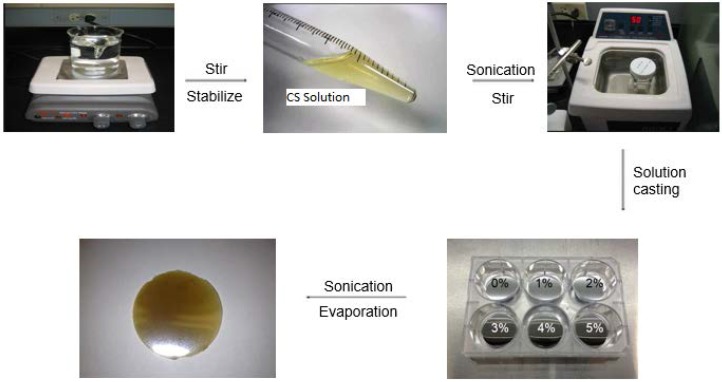
Schematic diagrams of the preparation process of CS and CS/ND films with various amount of ND ranging from 1 wt % up to 5 wt %.

A 2 wt % CS polymer solution was prepared by dissolving 2 g of CS powder in 100 mL 2% (v/v) acetic acid. A good dispersion was achieved by continuous stirring for 12 h on magnetic stirrer at temperature of 40 °C. CS/ND composites were prepared through a solvent casting method (Figure 1). A predetermined amount of as-received NDs (1% w/w of CS) and functionalized NDs (1%; 2%; 3%; 4%; 5% w/w of CS) was transferred into a 20 mL beaker containing 10 mL 2% v/v acetic acid aqueous solution. Then, the mixture was subjected to ultra-sonication bath treatment for 30 min. The ND suspension was then added to the pre-prepared CS solution (2 wt %) and stirred on magnetic stirrer at room temperature until no visible chunks could be observed. Surface polished stainless steel discs were pre-placed on the bottom of each well of a 6-well Petri dish for easy removal of the cast films. To completely eliminate the solvent, the blend liquid was dried in Petri dish at 35 °C for 48 h and sonicated repeatedly every 8 h during the evaporation. Films were obtained by peeling them off from the stainless steel discs and soaked in an aqueous solution of NaOH (0.4 wt %) at room temperature for 6 h to remove the residual acetic acid. Following repeated rinsing with deionized water, the samples were subsequently dried in vacuum and stored for further characterization.

### 2.4. Fourier Transform Infrared Spectroscopy (FT-IR)

FT-IR was used to confirm the carboxyl groups on ND surfaces. FT-IR spectra were recorded on a JASCO FT/IR-4100 spectrometer by KBr method (KBr: sample = 100:1, mass ratio). Samples were prepared by mixing 200 mg potassium bromide (KBr) with ~1.5 mg of ASND and FND, respectively. FT-IR spectra were recorded in the wavenumber range of 500–4000 cm^−1^ at a resolution of 2 cm^−1^.

### 2.5. X-ray Diffraction (XRD) Analysis

To determine the crystalline structure of the CS/ND nanocomposites, a Rigaku (Rotaflex Ru-200) X-ray diffractometer with a Co Kα line radiation resource was used. CS/ND nanocomposites were collected and mounted on the stage and analyzed in the scanning range from 5° to 70° in increments of 0.05° at a step size of 2 sec/step. The operation voltage and current of the X-ray source was 35 kV and 30 mA, respectively.

Detailed crystallinity is determined under the assumption that the areas are proportional to the scattering intensities of crystalline and amorphous phases [35]. Thus, the diffraction profile is separated into 2 parts: peaks are related to diffraction of crystallites, broad alone is related to scattering of amorphous, and the degree of crystallinity *X_c_* is measured as the ratio of crystalline area to total area.

(1) $$X_{c}=\frac{A_{cr}}{A_{cr}+KA_{am}}$$

where *X_c_* = degree of crystallinity; *A_cr_* = Area of crystalline phase; *A_am_* = Area of amorphous phase *K* = a constant related to the different scattering factors of crystalline and amorphous phases. For relative measures, *K* = 1.

### 2.6. Scanning Electron Microscopy (SEM)

The morphology of the composite samples was examined using Scanning Electron Microscopy (A JEOL JSM-6010LV SEM, Jeol Ltd., Tokyo, Japan) at 10 kV acceleration voltage. Samples were cut into 1 cm × 1 cm films and attached to the top of specimen holders by carbon tapes. All samples were coated with a thin layer of gold at a voltage of 1 kV for 20 s under vacuum with Gold Sputter Coater (Edwards S150B) prior to SEM characterization.

### 2.7. Differential Scanning Calorimetry (DSC)

The thermal properties including glass transition, degradation and interactions of CS/ND nanocomposites were studied using DSC (TA Instruments DSC Q100, New Castle, DE, USA) over a temperature range of 20–300 °C. Each specimen (~6 mg) was placed in sealed aluminum pans and heated under nitrogen atmosphere at a rate of 1 °C/min. DSC was calibrated with an indium standard. All specimen were kept in a desiccator to protect them from humidity prior to measurement. The glass transition temperature (*T*_g_) was obtained at the inflection point between the base lines where the heat capacity of the specimen changed.

### 2.8. Mechanical Property Characterization

The mechanical tests were performed from a micro-scale to nano-scale with the purpose of comparing improvement in the mechanical properties of CS/ND (functionalized and non-functionalized) samples to those of pure CS samples. Nano-indentation was employed to evaluate the nano mechanical properties including compression strength and modulus. In this work, nanoindenter manufactured by Center for Tribology, Inc. was carried out for the assessment of the micro/nano mechanical properties of CS/ND composites.

Composite films were cut into 5 mm × 5 mm squares and attached to the top of the metal mounts firmly with a very thin layer of glue, then transferred into a desiccator until the glue dried. In order to eliminate the thermal expansion and contraction during the experiment, all the specimens were placed on the stage inside the chamber of the nano-indentation instrument for thermal equilibrium for 3 h. To reduce the variation from the noise in laboratory conditions, a nanoindentation instrument was wrapped with soft cardboard with a 10 cm space in between, and all experiments were processed overnight. The indentation tests were performed with a strain rate of 0.03 s^−1^, and a maximum load of 50 mN. 25 (5 × 5) indents were applied for each type of sample. Load-displacement curves were plotted and manually corrected through UMT software. Young’s modulus and the hardness of each specimen were subsequently calculated and plotted. All the results were expressed as the mean value with a standard deviation.

## 3. Results and Discussion

### 3.1. Surface Functionalization of ND Particles Assessed by FTIR Spectroscopy

**Figure 2:**
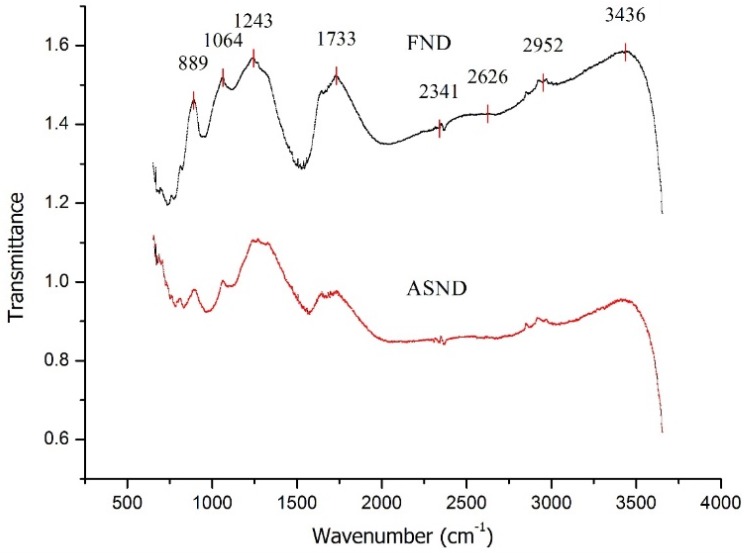
FTIR spectra of as-received ND (ASND) and functionalized ND (FND).

To provide evidence of functionalization of ND with acid, FTIR spectroscopy was performed (Figure 2) on as-received ND powders (AR-ND) and after functionalizing ND (F-ND). The peak at 3436 cm^−1^ can be attributed to the stretching vibration of surface –OH groups. The peaks at 2865 cm^−1^ and 2932 cm^−1^ are the characteristic peaks of ND, the stretching vibration of C-H bonding. The peaks around 1100–1400 cm^−1^ are probably caused by C–N and N–H bonds due to nitrogen impurity distributed in diamond structure and –C–O–C– absorption [36]. Those peaks are similar in intensity for both spectra, while the peak at 1733 cm^−1^ due to the stretching of –C=O groups is much higher in the spectrum of F-ND than in the spectrum of AS-ND, indicating that the acid treatment process produces additional –C=O functional groups on diamond surfaces.

### 3.2. Morphology of CS/ND Composites by SEM Spectroscopy

SEM was used to observe diamond agglomeration, particle size and distribution in the composites. ND particles have a strong tendency to aggregate in order to reduce the surface energy, which is a big challenge for synthesis of strong ND reinforced composites. In addition to van der Waals force, groups introduced by surface functionalization may result in inter-particle hydrogen bonding. To prevent agglomeration, many methods including mechanical attrition, sonication and surfactant introduction are commonly used [37,38]. In this research, sonication was used to facilitate the dispersion. 

**Figure 3:**
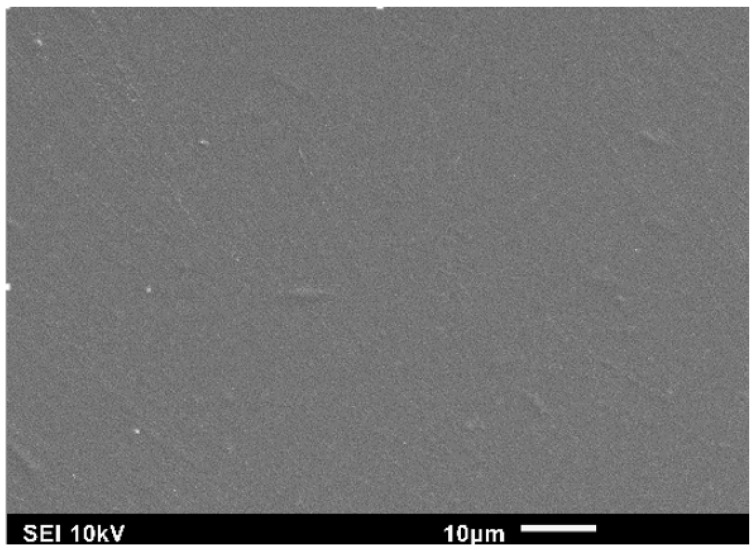
SEM images of pure CS.

**Figure 4:**
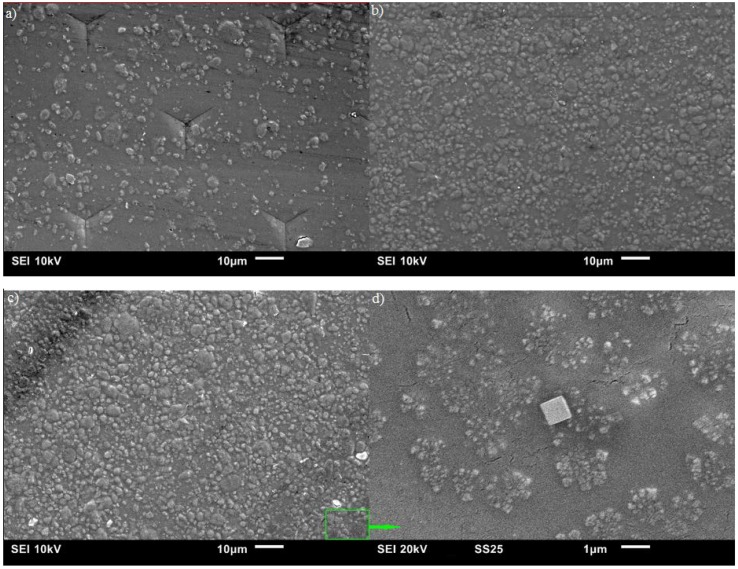
SEM images of CS with 1 wt % ND (**a**); 2 wt % ND (**b**); 4 wt % ND (**c**) and 4 wt % ND at higher magnification (**d**).

Figure 3 shows the surface morphology of CS, which is relatively flat and smooth compared to the composite samples with ND fillers as shown in the remaining five figures. All composite samples with ND ranging from 1 wt % to 4 wt %, shown in Figure 4, illustrate a random distribution of NDs at low magnification. At higher magnification, the sample with 4 wt % ND (see Figure 4d) shows a typical clustered random dispersion pattern, which means local agglomeration of ND fillers exists. We can clearly see that when the amount of ND fillers increases, particle density increases, the interparticle distance of ND particles decreases, and the particle size shows no significant difference. These results illustrate that the CS/ND nanocomposites fabricated by continuous ultrasonication have a relatively low agglomeration, which is critical for the composites to take advantage of the large surface area of nanoparticles to form a stronger interface for strengthening. In Figure 4a, no cracks or microscale defects can be observed in the composites after nano-indentation, indicating a strong interfacial interaction and a strong strengthening potential. At higher magnification, ND clusters in the 0.8~1.5 µm range and ND particles (~0.1 µm) were observed as shown in Figure 4d.

### 3.3. Characterization of CS/ND Composites by XRD 

**Figure 5:**
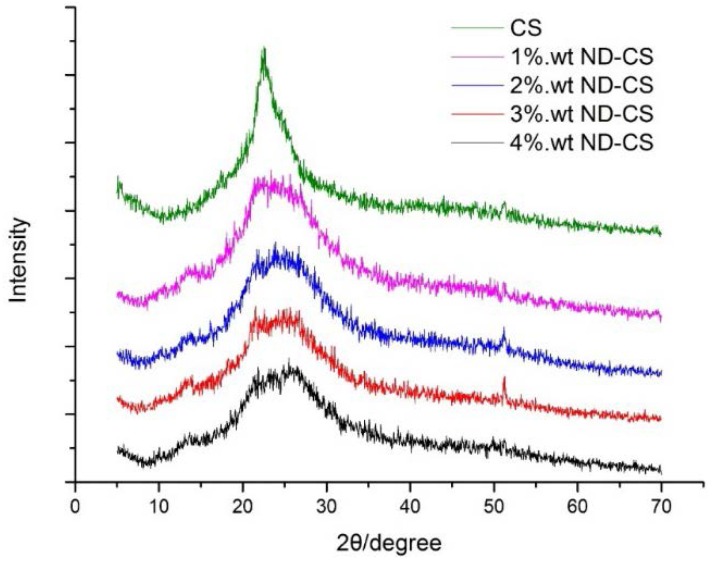
XRD patterns of pure CS and CS/ND composites with various ND concentrations.

To identify the crystalline structure change of CS and how ND influences the crystallinity of CS composites, XRD was carried out on the samples. The XRD patterns of all specimens are shown in Figure 5. All samples show the broad nature of the patterns which reveals that the CS and CS/ND composites are of a typical semi-crystalline structure [39]. There is one broad peak appearing in all the patterns. This peak is at approximately 22° and can be an overlap of (0 2 0) planes of the hydrated crystalline and amorphous structure of CS [40]. Upon the addition of ND to CS matrix, a satellite peak at 51.8° (with cobalt target, λ = 0.178897 nm) appears, which is attributed to the diffraction of the (1 1 1) plane of diamond crystal (*d_hkl_* = 0.205 nm), consistent with a previous report (44.2° with copper target, λ = 0.15405 nm) [41]. Another two new peaks at 13° and 26° appear which could be attributed to the new phases due to the interaction of CS and ND. Of these two peaks, the intensity of the peak at 26° increases as ND content increases compared to the typical peak of CS at 22°, which also reveals that the new formed phase is a result of the interaction between the ND and CS matrix.

The degree of crystallinity *X_c_* for each sample was calculated and listed in Table 1. It has been noticed that, by introducing ND into CS matrix, the crystalline part of the peak at 22° and the newly formed peak at 26° increases, indicating increased crystallinity of CS/ND composites compared to neat CS. This can be explained by the enhanced nucleation of CS on ND particles, which might assist in the transformation of CS polymer chains from an amorphous state to a crystalline state.

By a continuous increase in the amount of ND fillers from 1 wt % up to 4 wt %, the degree of crystallinity of CS/ND drops from 0.29 to 0.24 (Table 1). It can be interpreted as the interactions between ND fillers and CS matrix might partially break CS hydrogen bonding which results in a reduction of the crystallinity of CS in the composites. A higher amount of ND provides more surface area for the interaction with polymer chains, thereby resulting in lower crystallinity.

### 3.4. Characterization of CS/ND Nanocomposites by DSC

**Figure 6:**
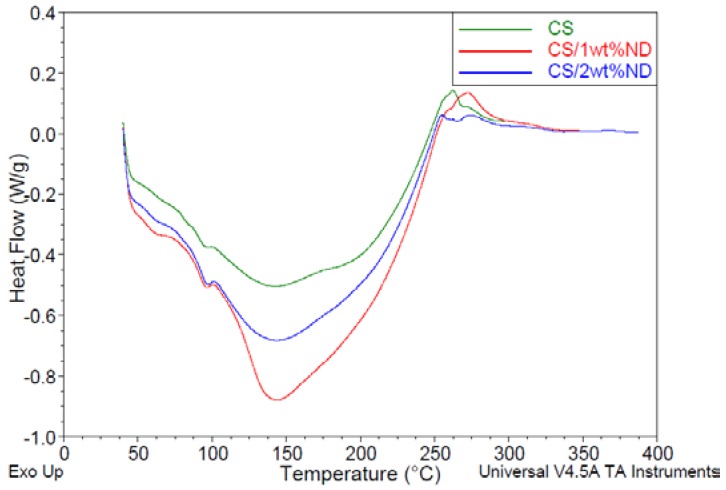
DSC Thermogram of CS, CS/ND composites with 1 and 2 wt % ND at a heating rate of 10 °C/min in nitrogen.

**Figure 7:**
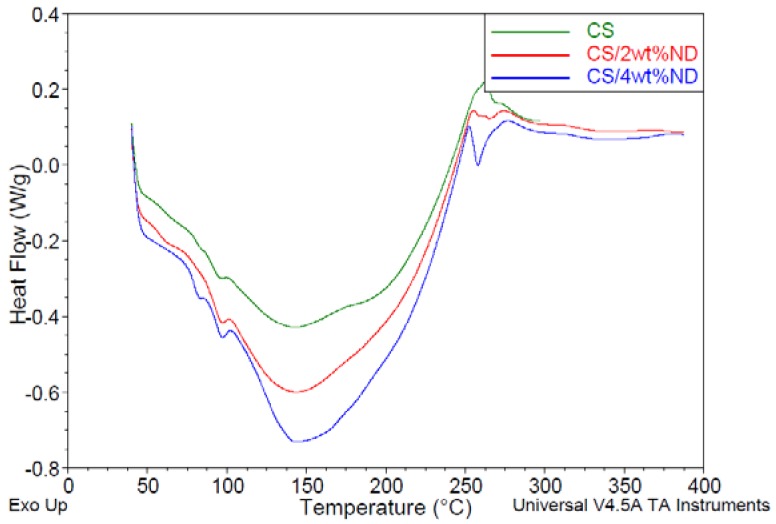
DSC Themogram of CS and CS/ND composites with 2 wt % and 4 wt % ND at a heating rate of 10 °C/min in nitrogen.

DSC analysis was conducted to understand the interaction between CS and ND. The DSC thermogram of CS (Figure 6) indicated exothermic transitions at 262.9 °C and 271.9 °C along with two endothermic transitions at 94.6 °C and 141.3 °C. The exothermic transition is an indication of CS degradation at 261.7 °C [42]. The occurrence of an endothermic transition at 94.6 °C could be attributed to the moisture present in the specimens or the loss of water associated with the hydrophilic groups of polymers. The presence of peak at 141.3 °C could be attributed to the interchain hydrogen bonding dissociation process of CS [42,43]. The glass transition temperature *T*_g_ was found at 174.9 °C for pristine CS, which is close to the reported value in the literature [38]. For DSC Thermogram of CS/ND with 1 wt % ND, the curve, shown in Figure 6, illustrates the moisture endothermic peak at 95.4 °C, hydrogen bonding dissociation at 142.4 °C, glass transition temperature at 180.4 °C and degradation temperature at 257.9 °C/273.2 °C, respectively. The hydrogen bonding dissociation temperature is consistent with previous literature in which the peak is around 180 °C [44,45]. DSC curves of CS/ND with 2 wt % ND and 4 wt % were obtained as well and are shown in Figure 7. The moisture endothermic peak, hydrogen bonding dissociation, glass transition temperature and degradation temperature of those two samples along with other samples are summarized in Table 2.

The endothermic peaks at around 95 °C associated with the loss of water from the hydrophilic groups of polymers varies from 95.4 °C to 97.0 °C for composite samples with ND, a little bit higher than the 94.6 °C obtained for CS, indicating that the structure change of CS/ND results in an energy increase for water to thermally evaporate.

*T*_g_ is an important parameter for the miscibility of polymer and fillers. Any physical property change in polymers due to filler incorporation can be reflected in *T*_g_. The raised glass transition temperature with increasing ND content could be explained by the fact that, with the incorporation of ND, the mobility of polymer chains was decreased and restricted due to the interaction of ND and CS chains. This result is consistent with the result of XRD in which the crystallinity was found to decrease.

By comparing the degradation temperature of various specimen, it is clearly observed that the thermal stability of second stage degradation increased with the addition of ND which can also be explained by the strong interaction between ND surface groups and polymer chains. For the first degradation process, a decreasing degradation temperature T_d_ could be explained by the fact that, in crystallized molecules of pure CS, they are arranged in a way where the interactions are the most strongest, when ND is introduced and interact with polymer chains, the molecules are moved away from their original position which may break the molecular symmetry, thus the formation of weaker interaction appears, and less energy is required to break them.

Detailed Enthalpy parameters are listed in Table 3. Enthalpy of loss of water increased from 1.158 J/g to 2.140 J/g when ND content increased to 4 wt %, illustrating that CS/ND composites have a higher water retention capability which is consistent with the results of moisture endothermic peaks. The endothermic enthalpy peak at approximately 145 °C increases significantly with the increase of ND content. This might be attributed to the hydrogen bonding resulting from the massive carboxyl, amino and hydroxyl groups introduced by ND as well as local partial melting of CS. As the hydrogen bonding occurs when a hydrogen atom bound to highly electronegative atom such as nitrogen and oxygen experiences attraction to other highly electronegative atoms. In this work, among hydroxyl groups and amino groups existing in both polymer and ND particles, more energy was required to break this electrostatic attraction.

Multiple peaks show in the curves ranging from 250 °C to 275 °C. The overlap peaks of CS can be roughly described as two exothermic degradation peaks. A new endothermic peak appears at around 256 °C for the CS/ND composites, as shown in Figure 5 and Figure 6. As the enthalpy of the new phase is positively associated with the amount of ND, it could be speculated that this new phase or structure with an endothermic thermal property might result from the interaction between ND and CS matrix. 

### 3.5. Mechanical Properties of CS/ND Composites

#### 3.5.1. Young’s Modulus

**Figure 8:**
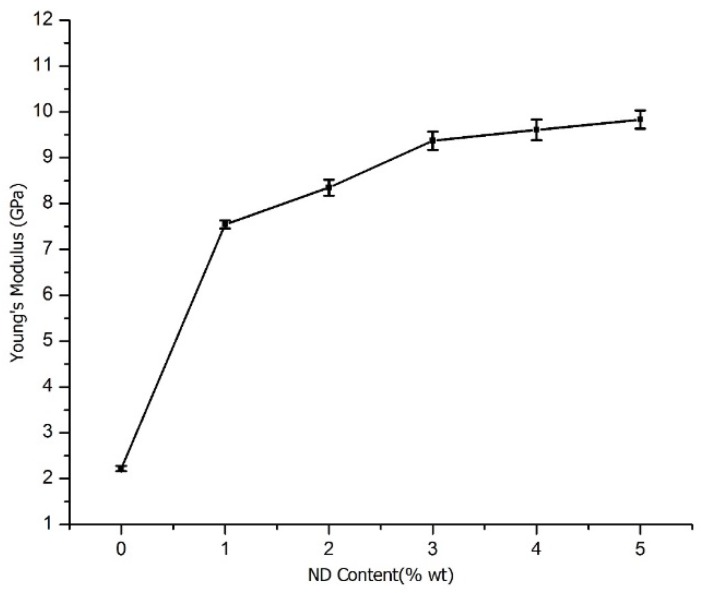
Young’s modulus of CS/ND plotted as a function of ND content.

Figure 8 shows the Young’s modulus of pure CS and CS/ND (functionalized) composites with a ND content ranging from 1% to 5%. The experimentally determined Young’s modulus of CS is 2.22 ± 0.06 GPa, which is very close to the values reported in previous literature [46]. The modulus values of the composites are much higher than pure CS and increase with the increase of ND content in the samples. The value for the CS/ND sample with 1 wt % ND is 7.55 ± 0.09 GPa, 2.4 times of that of pure CS. The value for CS/ND with 5 wt % ND is 9.83 GPa ±0.20 GPa, 3.4 times that of the pure CS.

Some analogous composites of polymer and nano-filler systems from previous literature have also been claimed to have increased Young’s modulus. It has been reported that ND-ODA/PLLA with a 5 wt % ND has a Young’s modulus of 5.9 ± 0.3 GPa, 2.27 times of that for the pure polymer matrix, measured by nano-indentation [47], whereas for PVA-ND with 0.6 wt % ND, the improvement of Young’s modulus was 98% to the PVA reference samples [48], and for epoxy/ND, the highest value of modulus is reported to be 6.12 ± 0.03 GPa at 4.0 wt % ND, 57% higher than that of the epoxy matrix [49]. Modulus loss was also found in some polymer/nano-fillers systems such as CS/Alg/nSiO_2_, modulus measured was decreased by 9% with the addition of 1 wt % nSiO_2_ from 8.99 ± 0.02 MPa to 8.16 ± 0.57 MPa [50].

Overall, the addition of ND into CS results in a great increment in Young’s Modulus. We attribute the results to two major factors: good dispersion and strong interaction between ND surface groups and CS matrix. A homogenous dispersion of ND provides large contact area to interact with the matrix. Functionalized surface groups on ND participate in the reaction with CS chains and cross link the particles and polymer matrix with hydrogen bonding to form a network structure, as suggested by DSC patterns, which could greatly hinder the chain’s movement and enhance the load transfer ability. This effect is illustrated by the significant Young’s modulus increase in CS/ND composites with 1 wt % ND. However, the increase of the modulus with ND content is not linear. This might be attributed to the increased ND agglomeration at higher ND content. Agglomeration would decrease effective area of ND to interact with polymer matrix. Besides, ND agglomeration form a network of ND particles with weak secondary bonding. These agglomerations are infiltrated by CS matrix, which is also the reason for structural instability. The improvement in Young’s modulus shown in Table 4 suggests that the Young’s modulus of the composites is strongly related to the degree of interaction between ND surface groups and polymer matrix, ND content, dispersion, and surface functional groups.

#### 3.5.2. Hardness

**Figure 9:**
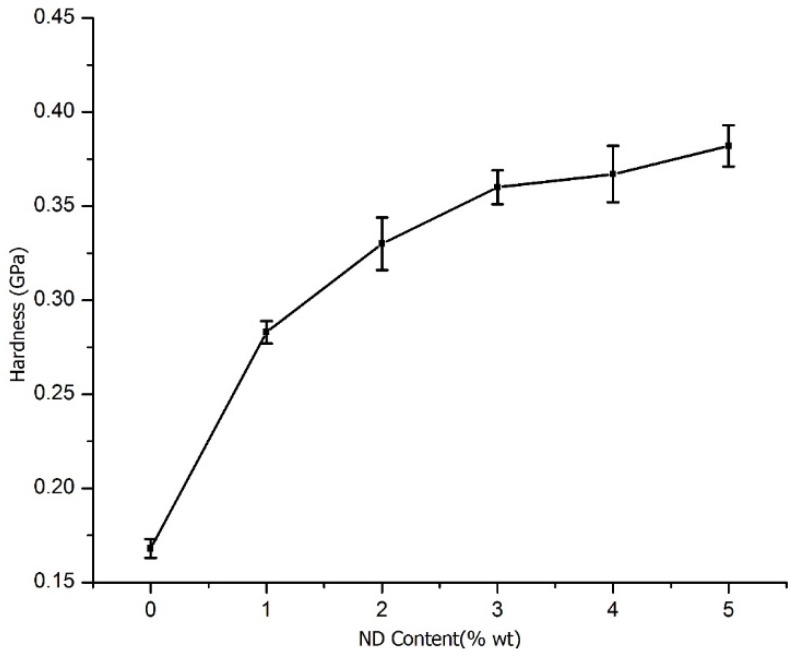
Hardness of CS/ND plotted as a function of ND content.

Hardness of CS/ND composites with ND content ranging from 0% to 5 wt % as measured by nanoindentation is shown in Figure 9. The hardness of the composites is much higher than CS and increases with the increase of ND content. Compared with the pure CS, the addition of 1 wt %, 2 wt %, 3 wt %, 4 wt %, 5 wt % ND increases the hardness by 68%, 96%, 114%, and 118%, and 127%, respectively.

To understand the effect of functionalization, CS/1 wt % as-received ND (AS-ND) composite was tested as reference. As shown in Table 4, the addition of as-received ND can also lead an increase in both Young’s modulus and hardness, however, the improvement attained is less than that with functionalized ND, suggesting that surface modification of ND is effective in enhancing interaction between ND and CS matrix and, thus, enhancing the strength and stiffness of the composites.

**Figure 10:**
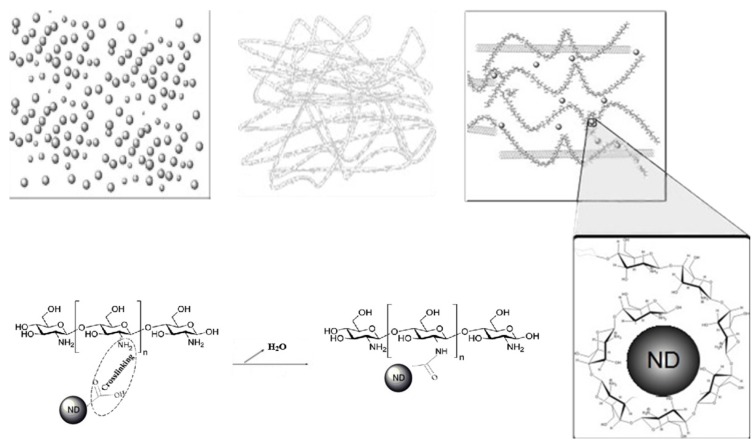
Hypothetical microstructure changes of ND/CS composites.

Figure 10 shows a hypothetical structure change because of the addition of ND, in which the surface groups of ND particles are surrounded and hydrogen is bonded to CS chains. This network structure is the key factor in enhancing mechanical strength by transferring load from ND particles to the CS matrix.

## 4. Conclusions

Synthetic biodegradable CS and ND composites were investigated for bone tissue scaffold application. The composite films were synthesized using a simple conventional method, solvent casting. The composites were then characterized by various advanced techniques to evaluate the structural, mechanical, and biomedical properties. SEM observation shows that ND particles in the composites disperse uniformly into the CS matrix and have good affinity with the CS matrix. XRD results suggest that the addition of ND to CS decreases the crystallinity of CS, which gives evidence to support the notion that ND with functional groups could interact with CS chains and form a more complex network. The mechanical properties of CS/ND composites measured by nanoindentation have been significantly improved with the addition of ND: 1 wt % ND had a 239% improvement in Young’s modulus and a 68% improvement in hardness. Also, the mechanical properties are positively correlated with ND content. With 5%ND, the Young’s modulus is increased by 3.4 times and the hardness is increased by 1.2 times. AS-ND is less effective in enhancing the mechanical properties of CS/ND composites than F-ND, indicating that surface groups on ND play important roles in enhancement of mechanical properties. DSC results show that the addition of ND can increase the glass transition temperature and the glass transition temperature increases with the increase of ND content, which further confirms the structural change of CS induced by the addition of ND. The biocompatibility and cytotoxicity evaluations are currently being carried out and the results will be reported in a future paper.

CS/ND composites exhibit the combined advantageous properties of CS and ND. The composites might “inherit” the biocompatibility and tunable biodegradability of CS as well as the high hardness and stiffness of ND, and are very promising for use as bone tissue scaffolds in tissue engineering.

## Figures and Tables

**Table 1:** Degree of crystallinity of CS/ND composites measured by XRD.

Sample Component	Degree of Crystallinity (*X_c_*)
CS	0.21
CS/1 wt % ND	0.29
CS/2 wt % ND	0.26
CS/3 wt % ND	0.24
CS/4 wt % ND	0.21

**Table 2:** DSC endothermic and exothermic peaks for CS and CS/ND composites.

Components/Peaks	Endothermic (°C)			Exothermic (°C)
CS	94.6	141.3	174.9	262.9/271.9
CS/1 wt % ND	95.4	142.4	180.4	257.9/273.2
CS/2 wt % ND	96.6	143.5	183.3	254.7/274.7
CS/4 wt % ND	97.0	149.9	187.5	253.9/276.5

**Table 3:** DSC Endothermic Enthalpy results for CS and CS/ND composites.

Component/Enthalpy	Exothermic Enthalpy (J/g)		
	Moisture	New Phase	Degradation
CS	1.158	–	0.213
CS/1 wt % ND	1.513	–	0.930
CS/2 wt % ND	2.266	1.831	1.331
CS/4 wt % ND	2.140	7.004	5.531

**Table 4:** Mechanical properties of CS/ND composites measured by nano-indentation.

Sample Composites	Young‘s Modulus (GPa)	Improvement (%)	Hardness (GPa)	Improvement (%)
CS/ 0 wt % ND	2.22 ± 0.06	0	0.17 ± 0.01	0
CS/ 1 wt % ND	7.55 ± 0.09	239	0.28 ± 0.01	68
CS/ 2 wt % ND	8.35 ± 0.17	276	0.33 ± 0.01	96
CS/ 3 wt % ND	9.37 ± 0.20	321	0.36 ± 0.01	114
CS/ 4 wt % ND	9.61 ± 0.23	333	0.37 ± 0.02	118
CS/ 5 wt % ND	9.83 ± 0.20	343	0.38 ± 0.01	127
CS/ 1 wt % ASND	7.14 ± 0.20	221	0.21 ± 0.01	26

Note: the errors are the standard deviation based on 20 measurements.
